# Amyloid β-Peptide Causes the Permanent Activation of CaMKIIα through Its Oxidation

**DOI:** 10.3390/ijms232315169

**Published:** 2022-12-02

**Authors:** Pol Picón-Pagès, Hugo Fanlo-Ucar, Víctor Herrera-Fernández, Sira Ausellé-Bosch, Lorena Galera-López, Daniela A. Gutiérrez, Andrés Ozaita, Alejandra R. Álvarez, Baldomero Oliva, Francisco J. Muñoz

**Affiliations:** 1 Laboratory of Molecular Physiology, Department of Medicine and Life Sciences, Universitat Pompeu Fabra, Calle Dr. Aiguader, 88, 08003 Barcelona, Spain; 2 Laboratory of Neuropharmacology, Department of Medicine and Life Sciences, Universitat Pompeu Fabra, 08003 Barcelona, Spain; 3 Cell Signaling Laboratory, Centro UC de Envejecimiento y Regeneración (CARE), Department of Cellular and Molecular Biology, Biological Sciences Faculty, Pontificia Universidad Católica de Chile, Santiago 8331150, Chile; 4 Laboratory of Structural Bioinformatics (GRIB), Faculty of Health and Life Sciences, Universitat Pompeu Fabra, 08003 Barcelona, Spain

**Keywords:** Alzheimer’s disease, amyloid, CaMKIIα, oxidative stress, CREB

## Abstract

Alzheimer’s disease (AD) is characterised by the presence of extracellular amyloid plaques in the brain. They are composed of aggregated amyloid beta-peptide (Aβ) misfolded into beta-sheets which are the cause of the AD memory impairment and dementia. Memory depends on the hippocampal formation and maintenance of synapses by long-term potentiation (LTP), whose main steps are the activation of NMDA receptors, the phosphorylation of CaMKIIα and the nuclear translocation of the transcription factor CREB. It is known that Aβ oligomers (oAβ) induce synaptic loss and impair the formation of new synapses. Here, we have studied the effects of oAβ on CaMKIIα. We found that oAβ produce reactive oxygen species (ROS), that induce CaMKIIα oxidation in human neuroblastoma cells as we assayed by western blot and immunofluorescence. Moreover, this oxidized isoform is significantly present in brain samples from AD patients. We found that the oxidized CaMKIIα is active independently of the binding to calcium/calmodulin, and that CaMKIIα phosphorylation is mutually exclusive with CaMKIIα oxidation as revealed by immunoprecipitation and western blot. An in silico modelling of the enzyme was also performed to demonstrate that oxidation induces an activated state of CaMKIIα. In brains from AD transgenic models of mice and in primary cultures of murine hippocampal neurons, we demonstrated that the oxidation of CaMKIIα induces the phosphorylation of CREB and its translocation to the nucleus to promote the transcription of ARC and BDNF. Our data suggests that CaMKIIα oxidation would be a pro-survival mechanism that is triggered when a noxious stimulus challenges neurons as do oAβ.

## 1. Introduction

Alzheimer’s disease (AD) is a neurodegenerative disease with the highest prevalence worldwide [1]. AD is a type of dementia whose main histopathological hallmarks are the presence of amyloid β-peptide (Aβ) forming extracellular senile plaques and intracellular fibrillary tangles caused by tau aggregation [2]. High concentrations of Aβ aggregate forming neurotoxic oligomers, initiating the damage in the hippocampus and causing memory loss [3,4].

The hippocampus is the centre in the brain for memory and learning based on glutamatergic neurotransmission. Glutamate released by the presynaptic terminal binds to both NMDA and AMPA receptors [5] at the postsynaptic terminal, allowing the entrance of sodium and calcium into the postsynaptic neuron. An increase in synaptic transmission will lead to a sustained calcium entry, which is necessary to induce long-term potentiation (LTP) [6]. Calcium entry will trigger an intracellular signalling initiated by the enzyme calcium/calmodulin dependent kinase IIα (CaMKIIα), that will lead to the phosphorylation and posterior nuclear translocation of the transcription factor cAMP response element-binding protein (CREB) [7]. This pathway is the main mechanism for learning and memory, promotes dendritic spine growth and is reported as being neuroprotective [8].

CaMKIIα is one of the four subtypes of CaMKII (-α, -β, -γ and -δ) [7]. CaMKIIα is a large protein (a homododecamer) activated by Ca^2+^/calmodulin binding, which induces its autophosphorylation. Autophosphorylation of one of the subunits facilitates Ca^2+^/calmodulin binding to the neighbouring units. This mechanism enables CaMKIIα to codify the intensity of calcium entry to produce different signalling outputs [9]. Furthermore, CaMKIIα autophosphorylated at T286 becomes active in a calcium-independent manner (autonomous CaMKIIα) [9]. This capacity allows for fine tuning of the intracellular signalling to regulate synaptoplasticity through CREB [7].

CaMKII has been reported to be susceptible to oxidation in cardiomyocytes resulting in pathological consequences in heart [10]. Oxidation affects methionines 281 and 282 in β-, γ- and δ-, while in CaMKIIα the oxidized residues are cysteine 280 and methionine 281. This oxidation happens only if CaMKIIα is previously activated by Ca^2+^/calmodulin. Once oxidized, cardiac CaMKIIα stays in a prolonged autonomous state with low kinase activity.

Since calcium entry is pathologically increased in AD due to the effect of Aβ oligomers (oAβ) on NMDA receptors [11], and oxidative stress is one the main neurotoxic mechanisms of Aβ [11,12,13], here we have aimed the study of the post-translational modifications of neuronal CaMKIIα due to oAβ and the effects on its activity.

## 2. Results

### 2.1. Oxidation Induces Autonomous CaMKIIα Activity

**Figure 1:**
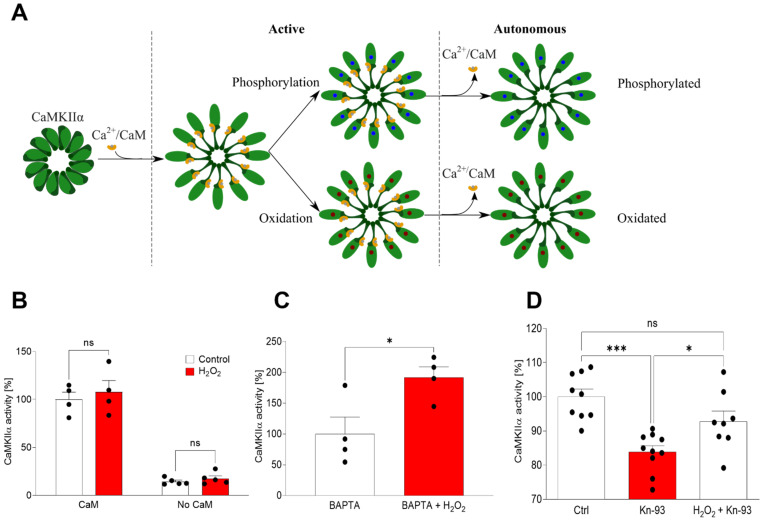
Oxidation induces a CaMKIIα autonomous state that is not inhibited by Kn-93. (**A**) Representation of CaMKIIα activation mechanism. After Ca^2+^/CaM binding the enzyme turns active by classical phosphorylation but also by oxidation. Later the Ca^2+^/CaM is released, and the enzyme continues to be active by oxidation in a state termed autonomous. (**B**) The activity of the enzyme was assayed in vitro by a commercial kit showing that 10 μM H_2_O_2_ is unable to activate CaMKIIα without Ca^+2^/CaM binding, neither is it able to affect the native activity of CaMKIIα. Data are the mean ± SEM of 5 independent experiments. non significant (ns) vs. the respective controls by two-way ANOVA plus Bonferroni as post-hoc test. (**C**) After initial activation by Ca^+2^/CaM, 10 μM H_2_O_2_ can induce an active state, which is independent of Ca^+2^/CaM as it was demonstrated by using 2 mM of the calcium chelator BAPTA-AM. Data are the mean ± SEM of 4 independent experiments. *p* < 0.05 vs. control by Student’s *t*-test. (**D**) In vitro analysis of the CaMKIIα activity showed that the activity of ox-CaMKIIα cannot be inhibited by 1 μM of the specific inhibitor Kn-93. Data are the mean ± SEM of 10 independent experiments. ns, * *p* < 0.05, *** *p* < 0.001 vs. the respective controls by ANOVA plus Bonferroni as post-hoc test.

It was previously reported that in cardiomyocytes ROS causes the pathophysiological activation of CaMKII by its oxidation [12,13], an activation state that occurs after Ca^2+^/CaM binding and lasts after the release of the Ca^2+^/CaM (Figure 1A). Here we have studied if this oxidation occurs in the α isoform in vitro (Figure 1B–D). Firstly, we demonstrated that CaMKIIα activity is dependent on the previous binding of Ca^2+^/CaM, since there was no activity when Ca^2+^/CaM was not preincubated with the enzyme (Figure 1B). Moreover, the oxidative stress inducer H_2_O_2_ did not affect the activity of CaMKIIα when it was bound to Ca^2+^/CaM (Figure 1B). Then, the activated enzyme was incubated with the calcium chelator BAPTA-AM previous to any contact with ATP, demonstrating that once the enzyme was oxidized it remained active even in the absence of Ca^2+^ [10,14] (*p* < 0.05) (Figure 1C). Finally, the incubation of the enzyme with the classical CaMKIIα inhibitor Kn-93 was able to reduce native CaMKIIα activity (*p* < 0.001), but not the activity of oxidized CaMKIIα (Figure 1D).

### 2.2. Aβ Triggers CaMKIIα Oxidation

**Figure 2:**
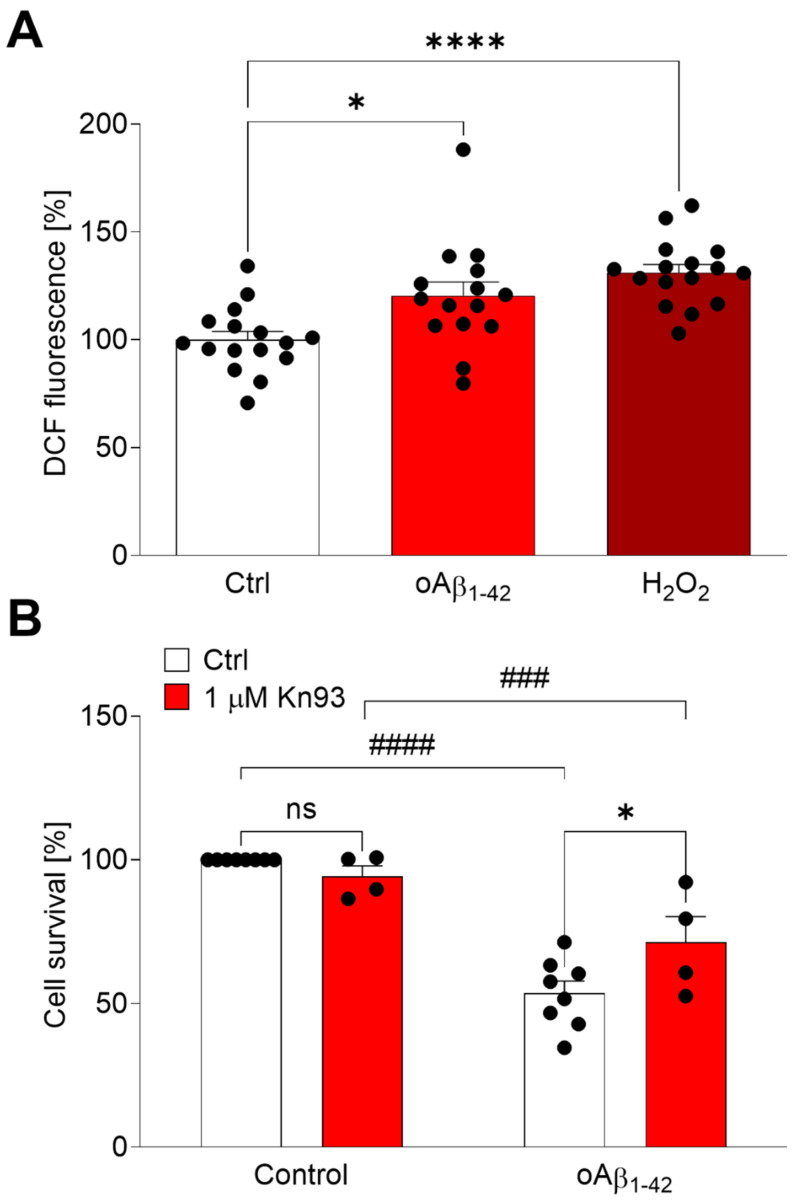
Kn-93 is able to induce protection regarding oAβ-mediated oxidative stress. (**A**) SH-SY5Y cells were treated with 10 μM oAβ_1–42_ or 200 μM H_2_O_2_ for 2 h. ROS production was measured quantifying the oxidation of H_2_DCFDA by the fluorescence emission at 522 nm. Data are the mean ± SEM of 8 independent experiments. * *p* < 0.05, **** *p* < 0.0001 vs. controls by ANOVA plus Bonferroni as post-hoc test. (**B**) SH-SY5Y cells were treated with 10 μM oAβ_1–42_ for 24 h with or without 1 μM Kn-93. Data are the mean ± SEM of 4–8 independent experiments. non significant (ns), ### *p* < 0.001, #### *p* < 0.0001 vs. the respective controls by ANOVA plus Bonferroni as post-hoc test.

Aβ induces neuronal death mostly by oxidative stress [11,15]. Therefore, we aimed the study on the effect of oAβ_1–42_ on neuroblastoma cells (Figure 2). Cells were treated with oAβ_1–42_ or H_2_O_2_, and the production of oxidative stress was analysed by the oxidation of H_2_DCFDA (Figure 2A). As expected, 10 µM oAβ_1–42_ and 200 µM H_2_O_2_ produced statistically significant amounts of free radicals (*p* < 0.05 and *p* < 0.0001, respectively). Furthermore, we studied the toxic effect of oAβ_1–42_ on neuroblastoma cells by MTT reduction assay (Figure 2B). We found that 10 µM oAβ_1–42_ was neurotoxic after 24 h of treatment (*p* < 0.0001) and this toxicity was partially reverted by the CaMKIIα inhibitor Kn-93 (*p* < 0.05), suggesting that a prolonged activation of CaMKIIα by oAβ_1–42_ could have deleterious effects.

**Figure 3:**
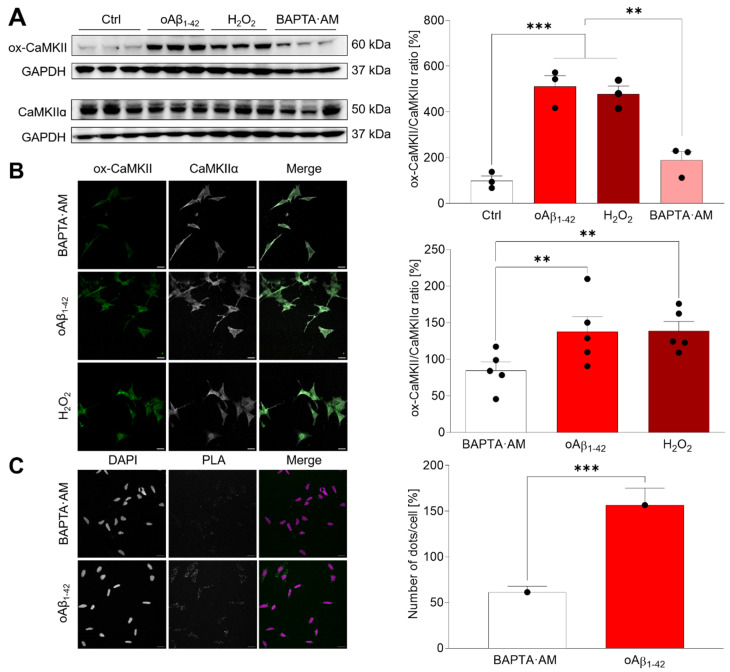
oAβ_1–42_ and H_2_O_2_ induce CaMKIIα oxidation. (**A**) SH-SY5Y cells were pre-treated for 30 min with 10 μM BAPTA-AM and later with 10 μM oAβ_1–42_ or 200 μM H_2_O_2_. Cell extracts were obtained and analysed by WB. A representative WB is shown. Oxidized and total CaMKIIα bands were normalized regarding GAPDH and the quantifications are shown in the graph. Data are the mean ± SEM of 3 independent experiment. ** *p* < 0.005, *** *p* < 0.001 vs. the respective controls by ANOVA plus Bonferroni as post-hoc test. The uncropped gels are shown in Figure S3. (**B**) SH-SY5Y cells were treated as explained in (**A**). Immunofluorescence experiments were performed with Abs against ox-CamKII and CaMKIIα. Results were analysed by confocal microscopy and representative images are shown. Fluorescence intensity was measured and expressed as the ratio between ox-CaMKIIa and the total enzyme in the graph. Data are the mean ± SEM of 5 independent experiments. ** *p* < 0.005 vs. the respective controls by ANOVA plus Bonferroni as post-hoc test. (**C**) The specific oxidation of the CaMKIIα isoform was studied by PLA assay. SH-SY5Y cells were treated with 10 µM oAβ_1–42_ for 30 min. A representative set of images is shown. The number of dots was quantified and normalized regarding the number of nuclei and shown in the right graph. Data are the mean ± SEM of 4 independent experiments. *** *p* < 0.001 vs. controls by Student’s *t*-test.

In order to study the effect of oAβ_1–42_ on CaMKIIα, we pre-treated SH-SY5Y cells either with or without 10 μM of the calcium chelator BAPTA-AM for 30 min, then we added either 10 μM oAβ_1–42_ or 200 µM H_2_O_2_, and cells were incubated for 30 min. Ox-CaMKIIα and the total CaMKIIα were studied by western blot (WB) (Figure 3). We found that both oAβ_1–42_ and H_2_O_2_ induce CaMKIIα oxidation (*p* < 0.001) (Figure 3A). The same results were obtained regarding the effect of oAβ_1–42_ and H_2_O_2_ on CaMKIIα oxidation when cells were studied by immunofluorescence (Figure 3B). Consistently, this oxidation was impaired when cells were pre-incubated with BAPTA-AM, which prevented the required activation by Ca^2+^/CaM, making it impossible to reach the autonomous state of activation (Figure 3A,B).

In order to demonstrate that within all CaMKII isoforms, the α isoform is able to become oxidized, we performed a PLA assay. This assay shows if two antigens recognized by two different antibodies (in this case anti-ox-CaMKII and anti-CaMKIIα) coexist within 50 nm, sharing a proximal location [16] (Figure 3C). Our results demonstrate that neuroblastoma cells treated with 10 μM oAβ_1–42_ for 30 min presented significant CaMKIIα oxidation (*p* < 0.001) (Figure 3C).

**Figure 4:**
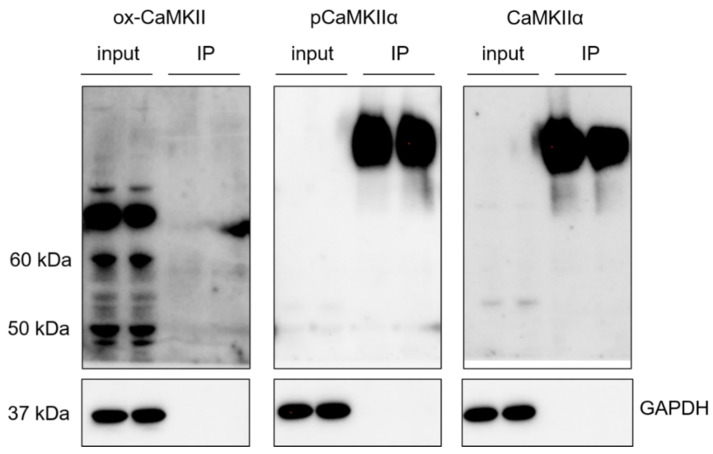
Phosphorylation and oxidation of CaMKIIα are mutually exclusive. SH-SY5Y cells were lysed, and the extracts were immunoprecipitated with an anti p-Thr286- CaMKIIα Ab. Then, immunoprecipitates were analysed by WB with Abs against pCaMKIIα, total CaMKIIα and ox-CaMKII. An input of the extracted protein was used as control, while GAPDH was used as control of the immunoprecipitation efficiency. WB did not show ox-CaMKII signal, meaning that oxidation was not occurring in the pCaMKIIα immunoprecipitates. A representative set of experiments is shown from 3 independent experiments. The respective negative controls and the WB obtained in the different experiments are shown in Figure S4.

Once it was demonstrated that CaMKIIα is oxidized by oAβ_1–42,_ we addressed the study of the relationship between both post-translational modifications, phosphorylation and oxidation. Cells were lysed, and phosphorylated CaMKIIα was immunoprecipitated in a proportion of 1:10 regarding the input (Figure 4). Multiple WBs were run to study the presence of total, phosphorylated and oxidized CaMKIIα, demonstrating that both modifications are mutually exclusive.

**Figure 5:**
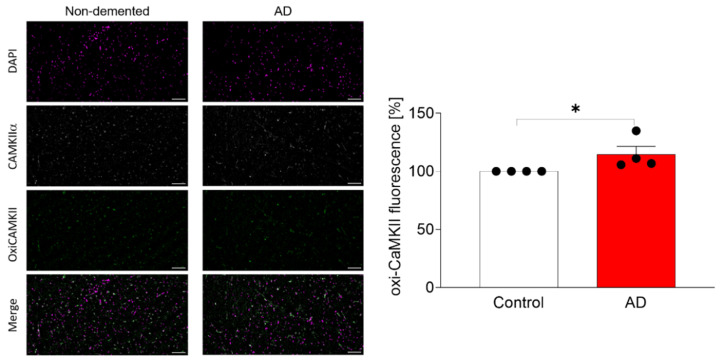
Study of the oxidation of CaMKIIα in cortical brain samples from non-demented donors and AD patients. Immunofluorescence experiments were performed with Abs against ox-CamKII and CaMKIIα. Nuclei were stained with Dapi. Scale bar represents 100 µM. Results were analysed by confocal microscopy and representative images are shown. Fluorescence intensity was measured and expressed as the ratio between ox-CaMKII and CaMKIIα in the right graph. Data are the mean ± SEM of 3–4 independent experiments. * *p* < 0.05 vs. the non-demented donors by Student *t*-test.

The pathophysiological relevance of these findings was assayed by the study of the CaMKIIα in AD brain samples (Figure 5). We performed immunofluorescence analysis on cortical samples from AD patients compared to samples from non-demented donors. As expected, we found a significant increase in CaMKIIα oxidation in AD brain samples compared to non-demented donors (*p* < 0.05).

### 2.3. In Silico Modelling of ox-CaMKIIα

**Figure 6:**
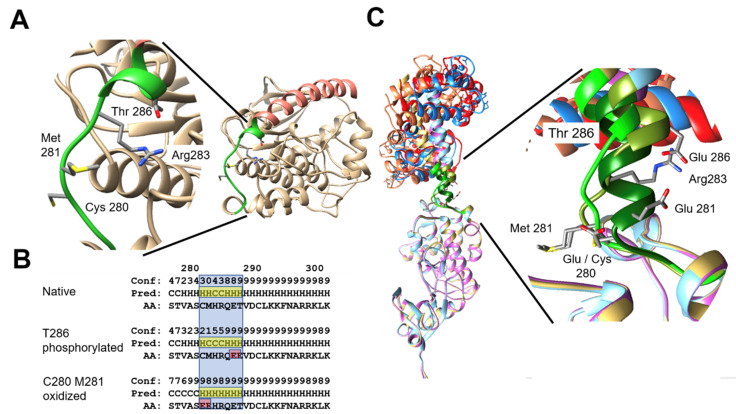
In silico study of the CaMKIIα oxidation. (**A**) The structural model shows a ribbon plate of Nt domain of CaMKIIα (yeast color), highlighting the a-helix calmodulin binding domain (in orange) and the loop region 277–288 (in green). Details of the loop conformation show the potential interaction between phosphorylated Thr-286 and Arg-283 to stabilize the helix capping [17]. (**B**) The bottom left panel shows the prediction of secondary structure (Pred) by PSIPRED of the amino-acids (AA) around 280–300 in the native sequence of CaMKIIα and the mutant forms T286E and C280E/M281E mimicking the phosphorylation and oxidation, respectively. The loop region is highlighted in blue, its predicted secondary structure in yellow and the glutamic acids resulting from the mutation in red. (**C**) Structural models of the wild-type and mutant forms of the Nt of CaMKIIα bound by calmodulin (yeast-orange for WT, cyan-blue for T286E and pink-red for C280E/M281E). Details of the conformation in the loop region 277–288 are shown in green (WT in light green, T286E in olive green and C280E/M281E in dark green).

We modelled the structure of CaMKIIα in silico and we also modelled the effect of putative mutants that mimicked the effect of the oxidation at C280 and/or M281 (Figure 6). An amplified detail of the loop shows the potential interaction between phosphorylated Thr-286 and Arg-283 in order to stabilize the helix capping (Figure 6A). We studied the effect of mutant isoforms by changing the phosphorylated and oxidized amino acids by glutamic (Figure 6B), which mimics the effect of such post-translational modifications resulting in the mutants T286E and C280E/M281E. The reliability of the prediction is scored from 0 to 9, 9 being the most reliable prediction (indicated as Conf). The prediction shows that phosphorylated T286 can help to sustain the helix (stabilization of the capping) despite extending the loop, while the oxidation of C280 and Met281 will extend the helix. We hypothesise that after oxidation of C280 and M281, the conformation of CaM-binding helix in the N-terminal domain of calmodulin-kinase will be less flexible, being more difficult to recover its original structure (Figure 6C). On the other hand, the phosphorylation of Thr286 will help to preserve the original conformation of the helix and even increase the flexibility of the hinge loop, helping to recover the original structure after the clamp of calmodulin releases the binding helix (Figure 6C).

### 2.4. Pathophysiological Effects of ox-CaMKIIα

**Figure 7:**
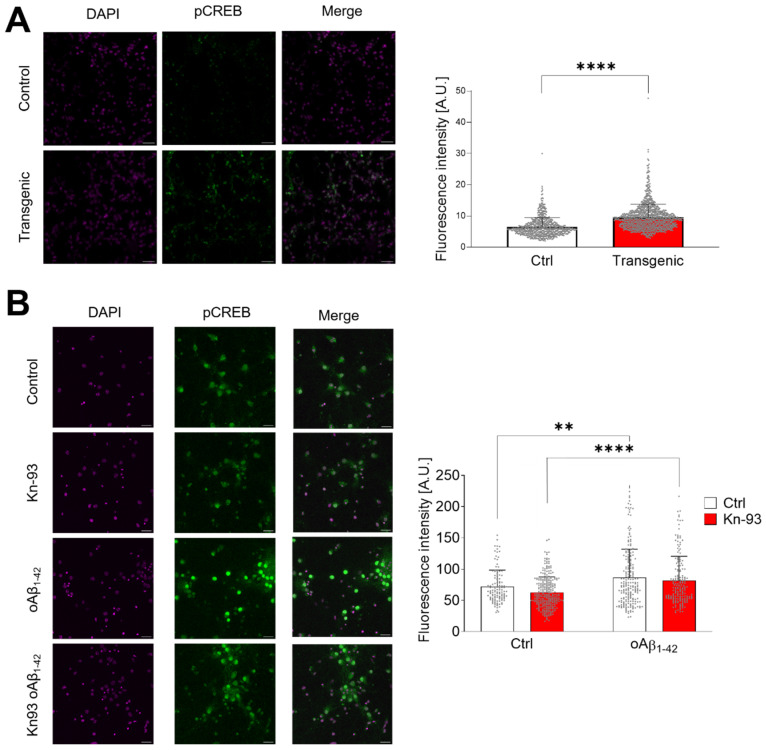
Oxidized CaMKIIα activates CREB. (**A**) Representative images of prefrontal cortex sections from 19-month-old mice analysed by immunofluorescence of p-CREB. DAPI was used for nuclei staining. Fluorescence of p-CREB was quantified and the data are shown in the right graph. Data are the mean ± SEM of 3 independent experiments. **** *p* < 0.0001 vs. controls by Student’s *t*-test. (**B**) Primary cultures of mouse hippocampal neurons were treated with 1 μM Kn-93 and 10 µM oAβ_1–42_ for 30 min. CREB phosphorylation was analysed by immunofluorescence with an Ab against p-S133-CREB. A representative set of images is shown. Fluorescence of p-CREB was quantified and the data are shown in the right graph. Data are the mean ± SEM of 6 different fields. ** *p* < 0.01, **** *p* < 0.0001 vs. controls by two-way ANOVA plus Bonferroni post-hoc test.

The CaMKII-CREB pathway is known to be protective for neurons since CREB phosphorylation and its nuclear translocation will start the transcription of pro-survival genes [18,19]. oAβ_1–42_ is a well-known inducer of neuronal toxicity [20,21,22] and one of the main mechanisms of neurotoxicity is the production of oxidative stress [23,24,25], which will promote the oxidation of CaMKIIα. Consistently, we have found that transgenic mice overexpressing APPswe/PSEN1dE9 (Figure 7A) have a significant higher expression of p-CREB than the cortical samples from wild type mice (*p* < 0.0001). The presence of Aβ deposits in the cortex of the mice is showed in Figure S1. Therefore, we have studied the role of oAβ_1–42_ on CREB phosphorylation (Figure 7B). Our results demonstrated that oAβ_1–42_ induced a prolonged activation of CREB by its phosphorylation (*p* < 0.01). This effect was independent of the phosphorylation state of CaMKIIα, since the inhibitor Kn-93 did not produce effects on the levels of p-CREB (*p* < 0.001).

**Figure 8:**
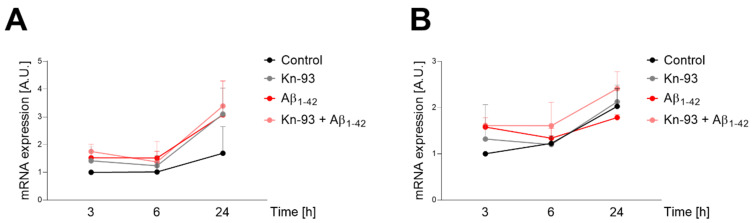
oAβ_1–42_ activates ARC and BDNF expression independently of the presence of Kn-93. (**A**) Real-time PCR quantification was performed for ARC expression in SH-SY5Y cells treated with 1 μM Kn-93, 10 μM oAβ_1–42_ or a combination of both during 3, 6 or 24 h. Data were normalized to controls at 3 h. Data are the mean ± SEM of 3 independent experiments. (**B**) Real-time PCR quantification for BDNF expression was performed and depicted as in (**A**). Data are the mean ± SEM of 3 independent experiments.

The downstream effect of CREB phosphorylation is the activation of the transcription of genes related with memory and learning and with neuronal survival [18,19]. For this reason, we have analysed by RT-PCR the transcription of ARC (Figure 8A) and BDNF (Figure 8B). In both cases (Figure 8), we found that the presence of Aß and the consequent oxidation of CaMKIIα increase the transcription of BDNF and ARC along 24 h independent of the presence of the CaMKIIα inhibitor Kn-93.

## 3. Discussion

Memory loss is a very early symptom of AD, which progresses to dementia. In particular, the hippocampus which is the centre of memory and learning, is dramatically affected by Aβ in AD. Therefore, we have studied the role of oAβ on the intracellular signalling that impairs memory formation. We focused our work in the post-translational modifications of CaMKIIα, the key enzyme that transmits the signal from the postsynaptic term to the soma allowing the translocation of CREB, a mechanism not just involved in memory formation but also in neuroprotection [26].

CaMKII oxidation has been studied previously in the heart, where it was found that it is pathological [10]. Consistently, we have found that CaMKIIα can be oxidized in vitro and this oxidized state of the enzyme results in permanent activity even in the absence of Ca^2+^. This state is termed “autonomous” since its activity becomes independent of Ca^2+^ once it is oxidized.

Therefore, our goal was to study of the effect of oAβ_1–42_ on CaMKIIα, since oxidative stress is playing a key pathophysiological role in AD [20]. oAβ_1–42_ is able to produce oxidative stress by itself [11,27], and here we have demonstrated that oAβ_1–42_ is producing free radicals that affect the cell viability of human neuroblastoma cells. The free radicals produced by oAβ_1–42_ oxidize CaMKIIα. This post-translational modification induces a CaMKIIα autonomous active state, like H_2_O_2_ does. The presence of ox-CaMKIIα in brain samples from AD patients show the pathophysiological relevance of the findings.

We have modelled the oxidation of CaMKIIα in silico to demonstrate that it produces the activation of the enzyme. This post-translational modification shows a different structural stability than CaMKIIα phosphorylation. As demonstrated by the structural studies that we performed, once Ca^2+^/CaM binds to CaMKIIα, the hinge structure that hides the catalytic domain opens. Both CM280-281 and T286 are located in the sequence that constitutes this hinge. Through CaMKIIα oxidation, this sequence becomes an α-helix, providing a structural stability that enables CaMKIIα to work in an autonomous way. Even though both CM280-281 oxidation and T286 phosphorylation stabilize the structure, they are mutually exclusive because their post-translational modifications share a common location in the α-helix. Furthermore, α-helix stability is lower in the oxidized state, which supports our enzymatic activity results. Finally, since oxidation remains for a longer time than phosphorylation, the final model depicts an ox-CaMKIIα that is mildly but constantly active compared to the phosphorylated CaMKIIα.

The capacity of sustaining an autonomous active state through phosphorylation enables CaMKIIα to change its conformational state and facilitate the phosphorylation of other monomers that constitute the physiological homododecameric CaMKIIα. This physiological effect is able to codify the intensity of neuronal activity and induce different kinds of signalling such as neuroprotection or the induction of LTP. Therefore, it is necessary to know if the effect of a pathologically driven oxidation of CaMKIIα is beneficial or detrimental for neurons. Since we found that CREB phosphorylation is increased in AD model mice and in hippocampal neurons challenged with oAβ_1–42_, we hypothesise that CaMKIIα oxidation would be a protective mechanism triggered by neurons after being challenged by oAβ_1–42_. This mechanism is a steady low active state, while phosphorylation is able to transduce potent calcium signalling [9,10,28,29]. This potent calcium signalling leads to an exponential threshold signalling necessary for the induction of LTP through phosphorylation of CREB at S133, S142 and S143 [30,31]. Thus, our results suggest a possible initial neuroprotective effect regarding the oxidative stress produced by oAβ_1–42_ through CaMKIIα oxidation to maintain CREB activity, which induces the transcription of genes involved in synaptic plasticity and survival such as ARC and BDNF, as we have found.

In conclusion, in this work we report the existence of CaMKIIα oxidation in AD brain samples and in neuroblastoma cells treated with oAβ_1–42_ as a model of AD. This oxidation generates a Ca/CaM autonomous state that may be beneficial regarding the beginning of Aβ neurotoxicity. However, due to the mutual exclusion of oxidation and physiological phosphorylation, this will lead to CaMKIIα becoming inactive. This inactivity can affect LTP and ultimately lead to neurotoxicity and cell death.

## 4. Material and Methods

### 4.1. Cell Lines

Human neuroblastoma cells (SH-SY5Y) were grown with Ham’s F12 medium plus GlutaMAX (Gibco, Waltham, MA, USA) supplemented with 15% fetal bovine serum (FBS; Biowest Nuaillé, France) and 1% penicillin/streptomycin (Gibco). Cells were incubated at 37 °C in a humidified atmosphere of 5% CO_2_. They were plated on 6-well plates (300,000 cells/well) for western blot (WB) studies or on 24-well plates with coverslips (90,000 cells/well) for immunofluorescence studies.

### 4.2. Primary Cultures of Mouse Hippocampal Neurons

Hippocampal neurons were isolated from 18-day-old CMB mouse embryos. This procedure was approved by the Ethics Committee of the Institut Municipal d’Investigacions Mèdiques-Universitat Pompeu Fabra. The hippocampi were aseptically dissected and tripsinized. Cells were seeded in DMEM high glucose (Gibco) supplemented with 10% horse serum and 1% penicillin/streptomycin into poly-D-Lysine coated plates (Gibco). After 120 min, the medium was removed and neurobasal medium (Gibco) was added containing 2% B27 supplement (Gibco), 1% penicillin/streptomycin, and 1% GlutaMAX (Gibco). On day three of culturing, cells were treated with 1.5 µM 1-ß-D-arabinofuranosylcytosine (Sigma, San Luis, MO, USA) for 24 h to eliminate proliferating non-neuronal cells. Hippocampal neurons were used for the experiments on day 15.

### 4.3. Human Cortical Brain Samples

Human cortical samples were supplied by the Neurological Tissue Bank of the Biobank-Hospital Clínic-IDIBAPS, Barcelona, Spain. The procedure was carried out according to the rules of the Helsinki Declaration and to the Ethics Committee of the Institut Municipal d’Investigacions Mèdiques-Universitat Pompeu Fabra (EC-IMIM-UPF). Samples were obtained from four non-demented control (two women and two men; 70 ± 0.8 years old) and three AD patient (two women and one man; 73 ± 1.8 years old).

### 4.4. Mouse Cortical Brain Samples

Mouse cortical brain samples were obtained from B6C3-Transgenic (APPswePSEN1ΔE9)85Dbo/J mice purchased from Jackson Laboratory (Bar Harbor, ME, USA) and wild-type mice. The procedure was carried out according to the rules of the Helsinki Declaration and to the Comité Ético Científico para el Cuidado de Animales y Ambiente (CEC-CAA) from Pontifical Catholic University of Chile. Samples were obtained from 19-month-old animals.

### 4.5. Aβ Preparation

1 mg of lyophilized Aβ_1–42_ (Anaspec, Fremont, CA, USA) was solubilized in 250 μL of sterile MiliQ water. The pH was adjusted to ≥10.5 with 1 M NaOH to avoid the isoelectric point of Aβ. Once dissolved, 250 μL of 20 mM phosphate buffer (pH 7.4) were added to the solution, followed by six 10 s-cycles of sonication (Bioruptor, Diagenode, Liège, Belgium). Finally, 2 μΛ of non-supplemented Ham’s F12 GlutaMax (Gibco) were added to the final 500 μL of disaggregated monomeric Aβ to achieve a concentration of 88.66 μM.

### 4.6. Aβ Oligomerization

Aliquots of monomeric Aβ were maintained at 4 °C for 24 h without agitation to allow its aggregation. After 24 h, they were frozen at −20 °C until use.

### 4.7. CaMKIIα Kinase Assay

CaMKIIα activity was measured by a commercial kinase activity kit (Promega, Madison, WI, USA). Briefly, 1 ng of CaMKIIα activity was assessed by the kinase ATP consumption kit (ADP-Glo Promega) and inferred from a consumption curve of 25 μM ATP/ADP at different ratios. The kinase was previously treated to induce its oxidation with 10 μM H_2_O_2_, free Ca^2+^ was chelated in some conditions using 2 mM BAPTA-AM (Millipore, Darmstadt, Germany) and the CaMKIIα inhibitor Kn-93 (MedChemExpress, Monmouth Junction, NJ, USA) was used at 1 μM. Then, the kit’s protocol was followed.

### 4.8. Reactive Oxygen Species (ROS) Production Assay

SH-SY5Y cells seeded in a 24-well plate (90,000 cells/well) were pre-incubated with 10 μM H_2_DCFDA (Life technologies, Carlsbad, CA, USA) in isotonic solution (140 mM NaCl, 2.5 mM KCl, 1.2 mM CaCl2, 0.5 mM MgCl2, 5 mM Glucose, 10 mM HEPES; pH 7.3–7.4 adjusted with Tris Base, osmolarity 300–310 mOsm adjusted with D-Mannitol) at 37 °C plus 5% CO_2_ and treated with either vehicle, 10 μM oAβ_1–42_ or 200 μM H_2_O_2_ for 2 h at 37 °C. Fluorescence was measured every three min at 522 nm by a VICTOR Nivo Multimade Plate Reader (PerkinElmer, Waltham, MA, USA).

### 4.9. MTT Reduction Assay

SH-SY5Y cells seeded in a 24-well plate (90,000 cells/well) were treated with vehicle, 1 μM Kn-93, 10 μM oAβ_1–42_ or a combination of both in FBS-free Ham’s F12 medium for 24 h. Then, cells were incubated with 0.5 mg/mL MTT (3-(4,5-dimethylthiazol-2-yl)-2,5-diphenyltetrazolium bromide) solution for 2 h. Formazan crystals were solubilized in 500 μL DMSO and cell viability was determined by absorbance at 595 nm by VICTOR Nivo Multimade Plate Reader (PerkinElmer).

### 4.10. Study of CaMKIIα Oxidation by WB in Neuroblastoma Cells

SH-SY5Y cells (6-well plates; 300,000 cells/well) were treated with 10 μM BAPTA-AM for 1 h or with either 10 μM oAβ_1–42_ or 200 μM H_2_O_2_ for 30 min in Ham’s F12 medium with 1% FBS. Then, cells were lysed on ice with 50 μL of RIPA buffer (150 mM Sodium Chloride, 1.0% Triton X-100, 0.5% Sodium Deoxycholate, 0.1% sodium dodecyl sulphate (SDS), 50 mM Tris, pH 8.0) supplemented with phosphatase and protease inhibitors (0.1 mM phenylmethylsulfonyl fluoride, 1 mM N-ethylmaleimide and 1 mM sodium orthovanadate). Cells were scraped and the samples were centrifuged at 15,000× *g* for 10 min at 4 °C. Samples were loaded into NuPAGE^TM^ Bis-Tris 4–12% protein gels. Next, proteins were transferred onto 0.2 μm pore nitrocellulose membranes. Membranes were blocked for 1 h at room temperature (RT) with 5% bovine serum albumin (BSA) in Tween 20-Tris buffer solution (TTBS: 100 mM Tris-HCl, 150 mM NaCl, pH 7.5 plus 0.1% Tween-20). Then, membranes were incubated overnight (o.n.) at 4 °C with rabbit anti-oxidized CaMKII (ox-CaMKII; 1:1000; GeneTex, Irvine, CA, USA), mouse monoclonal anti-CaMKIIα (1:1000; ThermoFisher, Waltham, MA, USA) or mouse anti-GAPDH (1:2000; Abcam) antibody (Ab). Horseradish peroxidase-conjugated donkey anti-rabbit and anti-mouse Abs (GE Healthcare, Chicago, IL, USA) were used as secondary Ab at 1:2000 for 1 h at RT. Primary and secondary Ab were diluted in 5% BSA in TTBS. Bands were visualized with Clarity Western ECL Substrate (BioRad, Hercules, CA, USA) and analysed with ImageJ software (version 1.53c).

### 4.11. Study of CaMKIIα Oxidation by Immunofluorescence in Neuroblastoma Cells

SH-SY5Y cells (24-well plates; 90,000 cells/well) were incubated with FBS-free Ham’s F12 with or without 10 μM BAPTA-AM for 30 min. Later, cells were treated with 10 μM oAβ_1–42_ or 200 μM H_2_O_2_ for 30 min. Cells were fixed with 4% PFA in PBS for 15 min, permeabilized with 0.2% Triton-100X in PBS for 10 min and then blocked with 5% BSA in PBS for 30 min. Coverslips were incubated o.n. at 4 °C in a humid chamber with 1:200 rabbit anti-ox-CaMKII (GeneTex) and 1:200 goat polyclonal anti-CaMKIIα (NovusBio, Centennial, CO, USA) Abs. Coverslips were incubated with Alexa Fluor 647 donkey anti-goat Ab (1:2000; Abcam), Alexa Fluor 488 donkey anti-rabbit Ab (1:2000; Invitrogen, Waltham, MA, USA) and DAPI (1:1000) for 1 h at RT. Coverslips were mounted onto slides with Fluoromount-G (SouthernBiotech, Birmingham, AL, USA). Digital images were taken with a Leica TCS SP5 Upright confocal microscope and analysed with ImageJ software (version 1.53c).

### 4.12. Study of CaMKIIα Oxidation by Proximity Ligation Assay

SH-SY5Y cells (90,000 cells/well) were treated with 10 μM BAPTA-AM for 1 h and 10 μM oAβ_1–42_ for 30 min. Cells were fixed with 4% PFA in PBS for 15 min and then permeabilized with 0.2% Triton-100X in PBS for 10 min. After these last steps, the Duolink^R^ Proximity Ligation Assay (PLA) Fluorescence Protocol established by Sigma-Aldrich (St. Louis, MO, USA) was followed. Primary Abs were 1:200 rabbit anti-ox-CaMKII and 1:200 goat polyclonal anti-CaMKIIα. 1:5 secondary Abs provided by the PLA kit (Sigma-Aldrich) were donkey anti-goat PLUS and donkey anti-rabbit MINUS. Digital images were taken with a Leica TCS SP5 Upright confocal microscope and analysed with ImageJ software (version 1.53c).

### 4.13. Immunoprecipitation and Study of CaMKIIα Phosphorylation and Oxidation

Twenty-four hours before the protein extraction from SH-SY5Y cells, we prepared sepharose beads for immunoprecipitation. Briefly, 100 μL sepharose G beads (Fisher Scientific, Hampton, NH, USA) were washed thrice with ice-cold TBS and centrifuged in between (3000× *g*; 3 min). Then, the beads were incubated o.n. with 1:50 rabbit anti-ox-CaMKII Ab (GeneTex) or 1:50 anti-phosphorylated-Thr286 CaMKIIα Ab (Thermo Fisher, Waltham, MA, USA) in ice-cold TBS with protease inhibitor cocktail (Roche Basilea, Switzerland) in rotation at 4 °C. Then, the beads were centrifuged at 3000× *g* for 3 min and blocked with 1% BSA in TBS for 1 h in rotation at 4 °C. Beads were washed again thrice with ice-cold TBS and kept at 4 °C while the procedure was carried out.

SH-SY5Y cells seeded in a 75 cm^2^ flask at 80% confluence were washed twice with PBS, trypsinized for 5 min and then centrifuged at 1200 rpm for 5 min. Pellets were lysed with 700 μL of non-denaturing lysis buffer (150 mM Sodium Chloride, 1.0% TritonX-100, 50 mM Tris, pH 8.0) supplemented with phosphatase and protease inhibitors (0.1 mM phenylmethylsulfonyl fluoride, 1 mM N-ethylmaleimide and 1 mM sodium orthovanadate), and centrifuged at 15,000× *g* for 10 min at 4 °C. 250 μL of extracted protein were added to the bead-Ab complexes and incubated in rotation for 4 h at 4 °C. Next, beads were washed again thrice with ice-cold TBS as before. To elute bead-attached proteins, 50 μL of 0.2 M glycine (pH 2–3) were added to the beads and incubated for 10 min with strong mixing at RT. Then, 50 μL of 1 M Tris-HCl (pH 7–8) were added, alongside 20 μL of loading buffer (concentrated five times). Samples were analysed by WB as described above, and samples reserved from the protein extraction were used as inputs.

### 4.14. Structural In Silico Modelling of CaMKIIα Oxidation

The structure of the N-terminus (Nt) domain of CaMKIIα was taken from PDB [32] (code 6W4O) and the conformation of the CaM binding helix was completed with the structure of AlphaFold2 [33] model (AF-Q9UQM7-F1_model_v3) up to residue 311. We used MODELLER [34] to model the structure of the Nt domain with the CaM binding helix bound by CaM. First, we removed the restrictions of the helix with respect to the Nt domain; then, we forced the contacts with CaM derived from the complex of a helix peptide with CaM (structure taken from PDB with code 6XXX). For the sequence of mutant T286E we used the model of the complex of the native sequence as the template, while for mutant C280E/M291E we added the restrictions of α-helix to the Nt capping (residues 280–285). PSIPRED [35] was used for the prediction of secondary structure using the full length of CaMKIIα/CaMKII.

### 4.15. Study of CaMKIIα Oxidation by Immunofluorescence in Human Samples

Human cortical sections (5 µm) were treated with 0.15 M glycine and 10 mg/mL NaBH_4_ (both in PBS) for 30 min. They were washed in deionized water, permeabilized with 0.3% Triton-100X in PBS for 1 h at 4 °C, and then blocked with 5% FBS + 1% BSA diluted in 0.1% Triton X-100 in PBS for 2 h. The following step was incubation o.n. at 4 °C with 1:200 rabbit anti-ox-CaMKII and 1:200 goat anti-CaMKIIα Abs. Samples were incubated the next day with Alexa Fluor 647 donkey anti-goat Ab (1:2000; Abcam), Alexa Fluor 488 donkey anti-rabbit Ab (1:2000; Invitrogen) and DAPI (1:1000) for 1 h at RT. They were then treated for 10 min with 4% Sudan Black (Sigma) in ethanol that was previously mixed o.n. at 4 °C, centrifuged at 3000× *g* for 20 min, filtered using common filter paper, washed with deionized water and mounted using Fluoromont. Digital images were taken with a Leica TCS SP8 confocal microscope and analysed with ImageJ software (version 1.53c).

### 4.16. Study of CREB Phosphorylation by Immunofluorescence in Murine Samples

Cortical brain samples were treated following the protocol described in Section 4.15. without using NaBH_4_. Then, samples were incubated o.n. at 4 °C with the primary Abs 1:400 rabbit anti-p-CREB (Cell Signaling, Danvers, MA, USA) and 1:100 mouse anti-Aβ (6E10, Covance, Princeton, NJ, USA). After washing the samples, they were incubated with Alexa Fluor 488 donkey anti-rabbit Ab (1:1000; Invitrogen), Alexa Fluor 555 donkey anti-mouse Ab (1:1000; Invitrogen) and DAPI for 1 h at RT. Digital images were taken with a Leica TCS SP5 confocal microscope and analysed with ImageJ software (version 1.53c).

### 4.17. Study of CREB Phosphorylation in Primary Hippocampal Neurons

Primary hippocampal neuronal culture was treated for 1 h with 100 nM oAβ_1–42_, 1 μM Kn-93 or a combination of both. Cells were fixed with 4% PFA in PBS for 15 min, permeabilized with 0.3% Triton-100X in PBS for 10 min and then blocked with 3% BSA in PBS for 30 min. Coverslips were incubated o.n. at 4 °C in a humid chamber with rabbit anti-pCREB (Ser133) (1:100; Cell Signaling) Ab. Coverslips were incubated with Alexa Fluor 488 donkey anti-rabbit Ab (1:2000; Invitrogen) and DAPI (1:1000) for 1 h at RT. Coverslips were mounted onto slides with Fluoromount. Digital images were taken with a Leica TCS SP5 Inverted confocal microscope and analysed with ImageJ software (version 1.53c).

### 4.18. Real Time PCR Analysis of Gene Transcription Due to CREB Phosphorylation

SH-SY5Y cells seeded in a 12-well plate (200,000 cells/well) were treated with either 1 μM Kn-93, 10 μM oAβ_1–42_ or a combination of both, in Ham’s F12 medium supplemented with 1% FBS, for 3, 6, or 24 h. Total RNA was isolated from cells using a NucleoSpin RNA isolation kit (Macherey-Nagel, Düren, Germany). SuperScript III reverse transcriptase system (Thermo Fisher Scientific) was used for complementary DNA synthesis. Quantitative real-time polymerase chain reaction was performed using SYBR Green (Applied Biosystems, Waltham, MA, USA) in a QuantStudio 12K Flex system (Applied Biosystems). GAPDH and HPRT1 were used as housekeeping genes for the quantification of relative gene expression using 2-ΔΔCt. Primers used for hCaMKIIα were commercially obtained from Sino Biological. We designed the other primers with the sequences indicated in Figure S2.

### 4.19. Statistical Analysis

Data are expressed as the mean ± SEM of the values from the number of experiments indicated in the corresponding figures. Student’s *t*-test, one-way analysis of variance (ANOVA) or two-way analysis of variance (ANOVA) plus Bonferroni as the post-hoc test were used for statistical analyses. *p* > 0.05 is considered not significant (ns).

## Data Availability

Not applicable.

## Supplementary Materials

The supporting information can be downloaded at: https://www.mdpi.com/article/10.3390/ijms232315169/s1.

## Abbreviations

A.U., arbitrary units; Ab, Antibody; Aβ, amyloid β-peptide; AD, Alzheimer’s disease; AMPAR, α-amino-3-hydroxy-5-methyl-4-isoxazolepropionic acid receptor; BSA, bovine serum albumin; CaMKII, calcium/calmodulin-dependent kinase II; Ca^2+^/CaM, calcium/calmodulin complex; CREB, cAMP response element-binding protein; FBS, fetal bovine serum; H2DCFDA, dichlorofluorescein; LTP, long-term potentiation; MTT, 3-(4,5-dimethylthiazol-2-yl)-2,5-diphenyltetrazolium bromide; NMDARs, N-methyl-D-aspartate receptors; Nt, N-terminus; ns, not significant; o.n., overnight; PBS, phosphate buffer saline; PFA, paraformaldehyde, ox-CaMKIIα, oxidized CaMKIIα; PLA, proximity ligation assay; RT, room temperature; WB, western blot.
