# Supplementary material for: Amyloid β-Peptide Causes the Permanent Activation of CaMKIIα through Its Oxidation
Source: Int J Mol Sci. 2022 Dec 2;23(23):15169. doi: 10.3390/ijms232315169 (PMC9740806; doi:10.3390/ijms232315169)
Supplement: Supplementary file 1 [file ijms-23-15169-s001.zip › ijms-1998378-supplementary.pdf]

Supplementary Materials

Figure S1

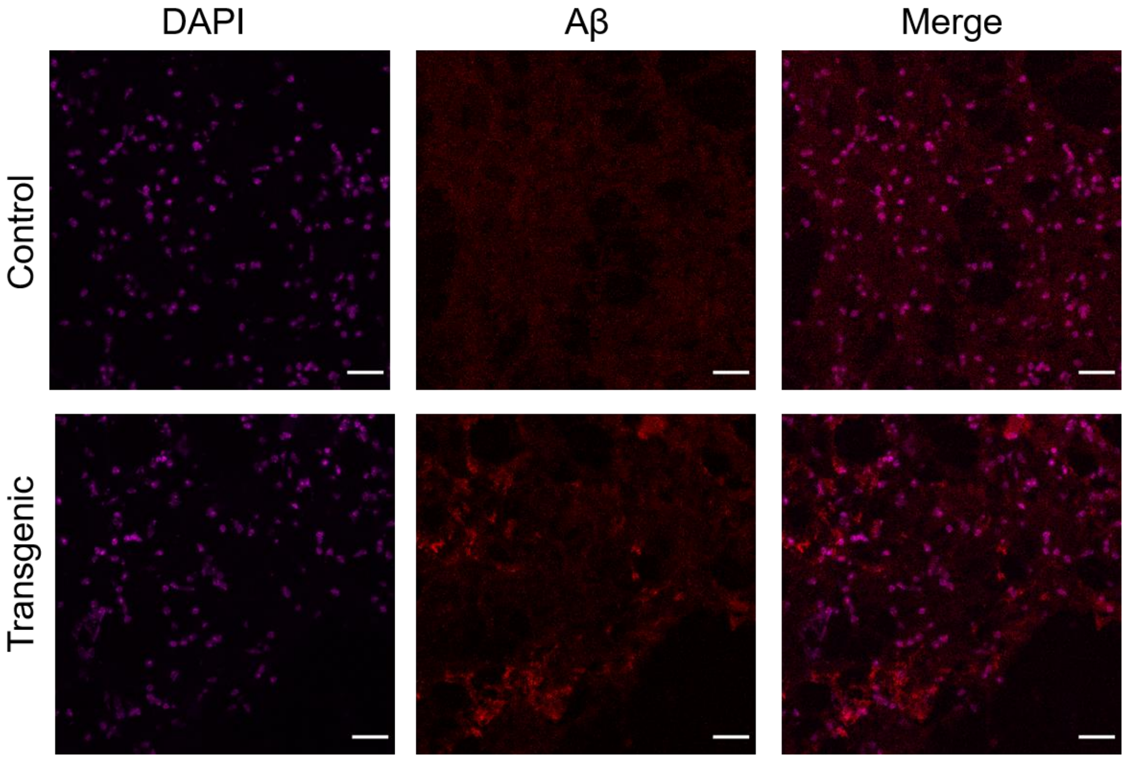

Figure S1. Representative images of the cortex from transgenic mice showing high Aβ deposits. Representative images of prefrontal cortex sections from 19-month-old mice. Immunofluorescence of Aβ (6E10; red fluorescence) was performed and DAPI (violet) was used for nuclei staining. The procedure was performed as explained in the section 4.16 of the article. Scale bar represents 25μm.

Figure S2

GAPDH

Forward: 5’-GGAGTCCACTGGCGTCTTC-3’

Reverse: 5’-TGGCTCCCCCCTGCAAATG-3’

HPRT

Forward: 5’-TGACACTGGCAAAACAATGCA-3’

Reverse: 5’-GGTCCTTTTCACCAGCAAGCT-3’

BDNF

Forward: 5’-AGTGCCGAACTACCCAGTCGTA-3’

Reverse: 5’-CTTATGAATCGCCAGCCAATTC-3’

ARC

Forward: 5’-AGCGGGACCTGTACCAGAC-3’

Reverse: 5’-GCAGGAAACGCTTGAGCTTG-3’

Figure S2. Sequence of the primers designed for RT-PCR studies.

Figure S3

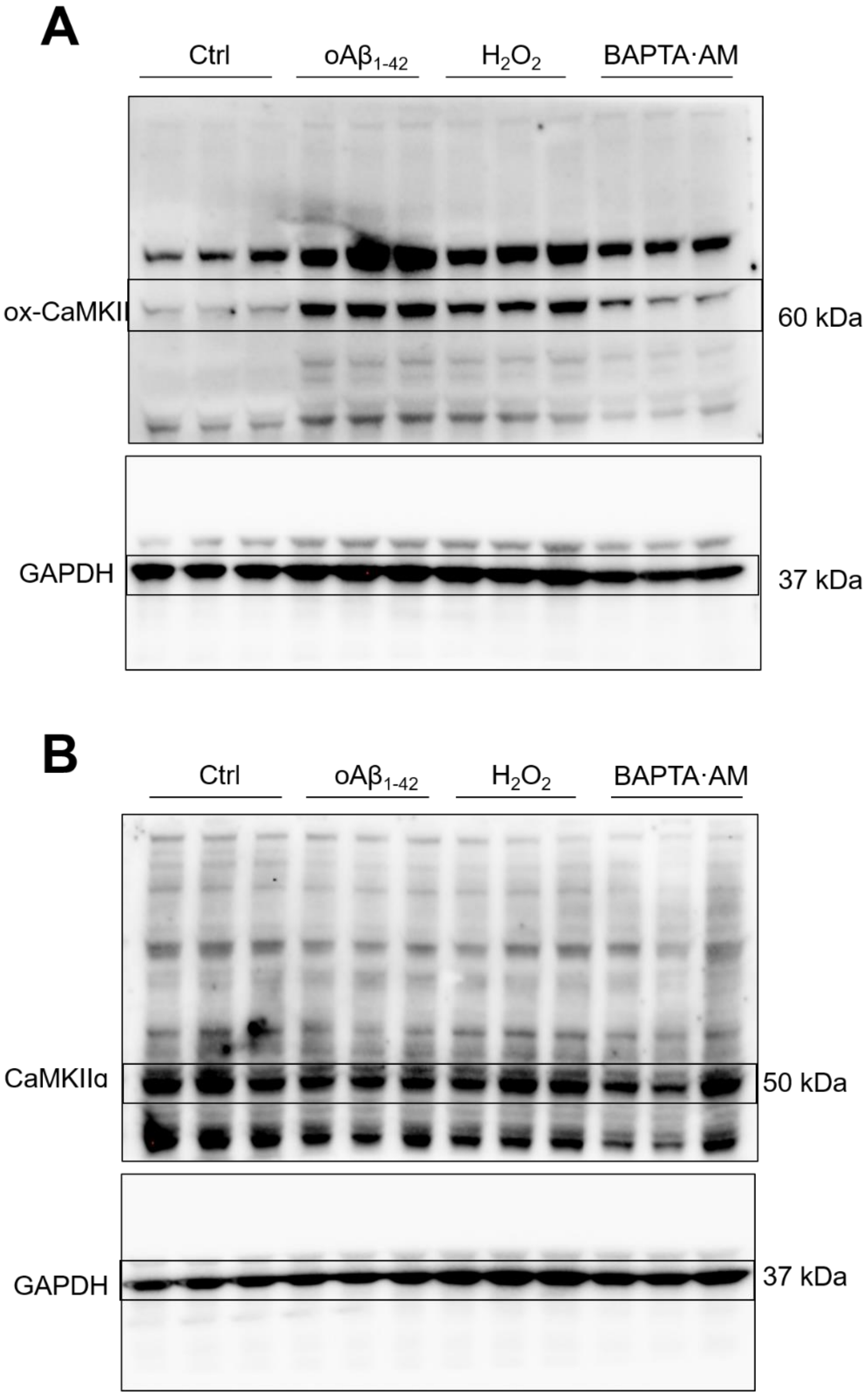

Figure S3. Uncropped WB membranes showed in the figures of the article. **(A)** WB of Figure 3A for ox-CaMKII. **(B)** WB of Figure 3A for CaMKIIa.

Figure S4

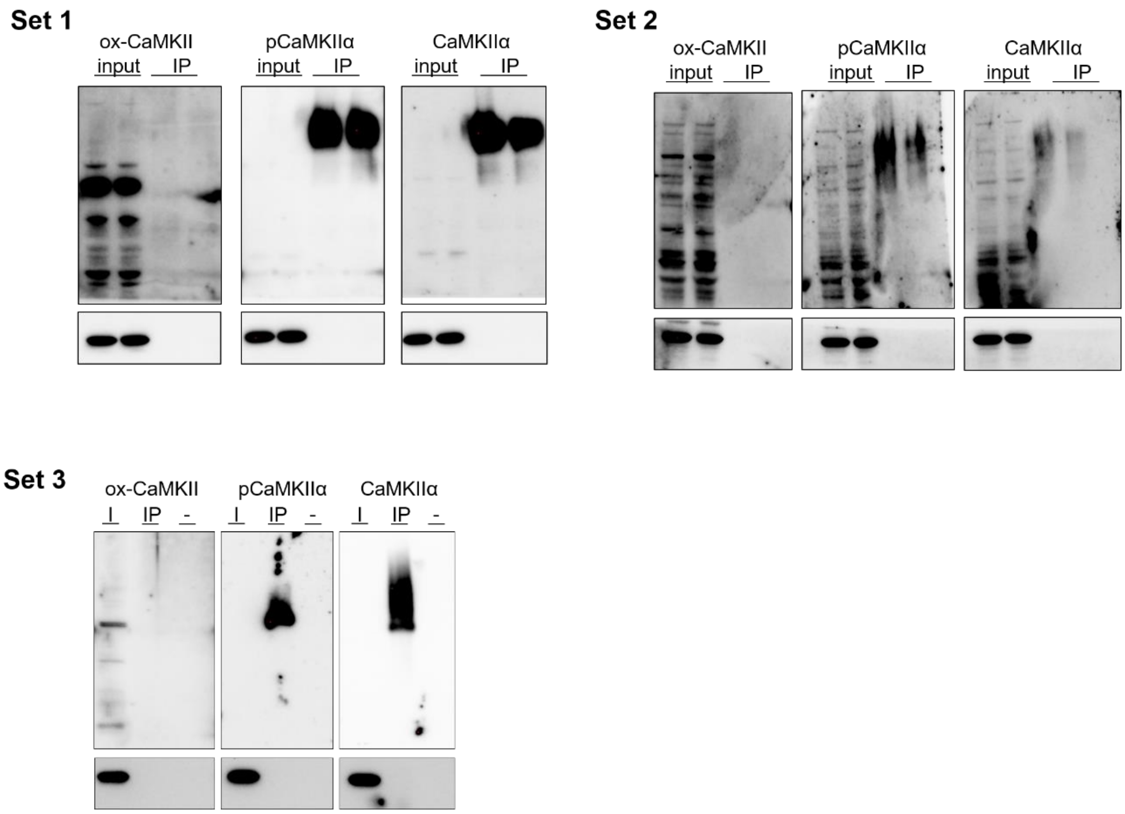

Figure S4. WB showing the immunoprecipitation of oxi- and p-CaMKII from Figure 4 plus two more independent experiments. A negative control (-) is also showed in the blot on the right.
